# Association of Recent Fatherhood With Antidepressant Treatment Initiation Among Men in the United Kingdom

**DOI:** 10.1001/jamanetworkopen.2023.16105

**Published:** 2023-05-31

**Authors:** Holly Christina Smith, Irene Petersen, Patricia Schartau

**Affiliations:** 1 Department of Primary Care and Population Health, University College London, Royal Free Campus, London, United Kingdom; 2 Epidemiology and Health Informatics, Department of Primary Care and Population Health, Institute of Epidemiology & Health, University College London, Free Campus, London, United Kingdom

## Abstract

**Importance:**

There is some evidence that men may be at higher risk of depression directly following the birth of their child and that approximately 1 in 10 men will experience depression in the year after birth, but less is known about men’s antidepressant treatment during this time. Likewise, few direct comparisons have been made with antidepressant treatment in men who have not recently become fathers.

**Objectives:**

To determine whether recently having a child was associated with increased odds of antidepressant treatment in men.

**Design, Setting, and Participants:**

This cohort study used UK primary care electronic health records from the IQVIA Medical Research Database. Participants included men aged 15 to 55 years who had had a child in the previous year, from January 2007 to December 2016, and compared with up to 5 men who did not have a child in the same calendar year. Data were analyzed from January 2022 to March 2023.

**Exposure:**

A record of having a child in the previous year was identified through linked primary care records using a family identification number.

**Main Outcomes and Measures:**

The main outcome was antidepressant initiation in the year after childbirth or in the year after this index date for men who did not have a child. Random-effects Poisson regression was used to determine associations of cohort, age group, social deprivation, history of antidepressant treatment, and calendar year with having an antidepressant prescription in the year after index date using prevalence risk rates (PPRs).

**Results:**

Analysis included 90 736 men who had had a child in the previous year and 453 632 men in the comparison cohort. Most men in the study (463 879 men [85.2%]) were aged between 25 and 44 years, and there were more men living in the least deprived areas (130 277 men [23.9%]) than the most deprived areas (72 268 men [13.3%]). Overall, 4439 men (4.9%) had at least 1 antidepressant prescription in the year after they had a child, compared with 26 646 men (5.9%) who did not have a child in the same year. However, after adjustment there was no difference in antidepressant treatment between groups (adjusted PRR [aPRR], 1.01; 95% CI, 0.98-1.04). In fathers, those who had recently received antidepressant treatment were much more likely to receive antidepressant treatment after childbirth compared with fathers with no history of antidepressant treatment (aPRR, 32.31; 95% CI, 30.37-34.38). Fathers living in the most deprived areas were 18% more likely to have an antidepressant prescription compared with fathers living in the least deprived areas (aPRR, 1.18; 95% CI, 1.07-1.30).

**Conclusions and Relevance:**

These findings suggest that recently having a child was not associated with an increase in antidepressant treatment among men, but previous antidepressant treatment in fathers was strongly associated with treatment after childbirth. Further research is needed to determine whether antidepressant treatment or experiencing depression can be a barrier to fatherhood and whether fatherhood is a barrier to receiving antidepressant treatment.

## Key Points

**Question:**

Is having a new child in the past associated with increased antidepressant treatment among men in the United Kingdom?

**Findings:**

This cohort study of 90 736 fathers and 453 632 men who did not have a child in the same year found no difference in antidepressant treatment between these groups once all factors, including history of treatment, were accounted for. However, history of antidepressant treatment was a key factor associated with further treatment among men who had a child in the past year.

**Meaning:**

These findings suggest that recent fatherhood was not associated with an increase in antidepressant treatment.

## Teaser

This cohort study assesses the association of recent fatherhood with antidepressant treatment compared with men who did not have a child in the previous year.

## Introduction

It is estimated that depression affects approximately 3 in 100 men aged between 16 and 55 years in England,^1^ and the rate of antidepressant treatment is approximately 1 in 100 person-years for men aged between 18 and 39 years in the United Kingdom (UK).^2^ There is some evidence that men may be at higher risk of depression and other common mental disorders directly following the birth of a child,^3,4,5^ as the first year after childbirth (postnatal period) is a time when many parents experience changes in their social, emotional and relationship roles, are adjusting to being a parent, and experience additional work and financial stress.^3,6,7^ There is also growing evidence that there is a link between maternal and paternal mental health.^8^

Studies estimate that 8.8% of men experience depression during the postnatal period,^9^ but it is less clear whether fatherhood is associated with antidepressant treatment and whether men are more likely to need antidepressant treatment following the birth of a child than at any other time of their life. In this study, we used UK primary care electronic health records (EHR) to conduct a cohort study of fathers’ antidepressant treatment in the year after having a child compared with men who did not have a child in the same time period.

## Methods

This cohort study was approved by the IQVIA Scientific Review Committee. IQVIA Medical Research Data (IMRD) incorporates data from THIN (previously The Health Improvement Network), a Cegedim Database. Reference made to IMRD is intended to be descriptive of the data asset licensed by IQVIA. The requirement for informed consent was waived because this study uses deidentified data provided by patients as a part of their routine primary care. As our data are anonymized, it is not possible to disseminate our findings to study participants. We discussed the broad concept of this study with a small group of parents who helped us refine our study plans and the plain English summary. This report follows the Reporting of Studies Conducted Using Observational Routinely-Collected Health Data (RECORD) statement reporting guidelines.

### Setting

In the UK, health care is free at the point of delivery for all residents as part of the National Health Service. Primary care is typically the first point of contact for anyone experiencing problems with their mental well-being in the UK. Care is largely delivered by general practitioners (GPs) who can identify depression and initiate treatment, including antidepressant treatment. Information about patients and their health and treatment are collected during consultations. This information is primarily used for clinical care but is also widely used for research through large health care databases.

### Data Source

We used a UK primary care database, IMRD, incorporating data from THIN, a Cegedim database. As of December 2016, IMRD contained anonymized electronic health records for 16 million registered patients from 730 practices across the UK.^10^ The database contains patient-level information on demographics, prescribing, symptoms, procedures, prevention, lifestyle factors, and diagnostics. Prescribing information is categorized according to the British National Formulary (BNF) classification using BNF codes.^11^ Socioeconomic information is captured through the Townsend score, which provides a measure of material deprivation based on where a person lives, unemployment, car ownership, home ownership, and household overcrowding.^12^ Scores range from 1 to 5, with higher score indicating more deprivation. IMRD is broadly representative of the UK population in terms of demographics, chronic disease, and mortality; however, there is an overrepresentation of more affluent people in the IMRD.^13^ Data quality criteria were applied whereby practices that did not have acceptable computer use^14^ or acceptable mortality rates^15^ by the date of childbirth were excluded.

### Study Population: Fathers

In IMRD, we identified men aged 15 to 55 years with a record of having a newborn child between January 1, 2007, and December 31, 2016. This was identified through the records of cohabiting mothers and newborns via their household identification number. This method has been used previously to identify potential fathers in electronic health records.^5^ We applied a range of inclusion and exclusion criteria based on the age of the father and mother and the household composition eAppendix in Supplement 1).

### Study Population: Comparison Cohort

For each father in the study, we selected up to 5 men who had no record of having a newborn child within the same calendar year, matching on GP practice and men’s year of birth. Men in this cohort were assigned the same index date and follow-up period as the fathers (ie, the date of their child’s birth) they were matched to. These men had to meet the same data quality criteria as the fathers at their index date to be included in the study. They had to have 1 complete year of follow-up information from index date and have complete social deprivation information. Men were sampled with replacement and as such could be in the comparison cohort for multiple fathers in the study.

### Definition of Variables

#### Primary Outcome: Antidepressant Treatment

Men were followed up for 12 months after their index date, and they were considered to be treated with antidepressants if they had a record of an antidepressant prescription in that time period. Antidepressants were identified within a man’s therapy record and included selective serotonin reuptake inhibitors, tricyclic and related antidepressant drugs, monoamine-oxidase inhibitors, and other antidepressant drugs, such as mirtazapine. These were identified as any prescription categorized under BNF chapter 4, subchapter 3.^16^ Any prescriptions for amitriptyline or duloxetine were excluded, since they likely related to treatment for pain or anxiety rather than depression. Participants were considered at risk of exposure from index date up to the earliest of either their first antidepressant prescription or the end of 1 year of follow-up.

#### Patient Characteristics

We stratified our analysis by paternal age, social deprivation (Townsend score), history of antidepressant treatment, and calendar year. Men were assigned to a 5-year band according to their age. Using Townsend scores, each man was assigned to 1 of 5 groups of deprivation, from least to most deprived. History of antidepressant treatment was categorized as: recent treatment, indicating those who had a record of any antidepressant treatment in the period of 1 year before date of childbirth (or equivalent index date) up to 1 day before date of childbirth; previous treatment, those who had a record of any antidepressant treatment in the period of 2 years before date of childbirth (or equivalent index date) up to 1 year and 1 day before date of childbirth; and none, those with no record of any antidepressant treatment in the 2 years prior to date of childbirth. If a person had both previous and recent treatment, they were categorized as having recent treatment only. An exploration of using different time periods to categorize history of antidepressant treatment was conducted and is included in eTable 1 in Supplement 1. Calendar year was grouped into 2-year bands.

### Statistical Analysis

For the primary outcome measure, the proportion of men with an antidepressant prescription in the follow-up period is given, comparing fathers with the comparison cohort, stratified by patient characteristic. The time to first prescription was estimated and given as the median (IQR) number of days. Random-effects Poisson regression was used to determine associations of age group, social deprivation, history of antidepressant treatment, and calendar year with having an antidepressant prescription in the year after index date in fathers only. Three models were created: unadjusted; adjusted for age, social deprivation, and year-group; and fully adjusted, including adjustments for age, social deprivation, history of antidepressant treatment, and year group. These estimates are presented as prevalence risk rates (PPRs) and 95% CIs. To account for clustering by GP practice, they were included as a random-effects (random intercept) term. This analysis was repeated including the comparison cohort and cohort group as a variable. No interaction terms were included in the models.

All analyses were conducted in January 2022 to March 2023 using Stata statistical software version 16 (StataCorp). Statistical significance was determined by 95% CIs that did not cross 1.

## Results

### Participants

Between January 1, 2007, and December 31, 2016, we identified 90 736 fathers who met our inclusion and exclusion criteria (Figure). Matching for GP, age, and calendar year identified 453 632 men in the comparison cohort with no record of having a newborn child in their index year.

**Figure.**
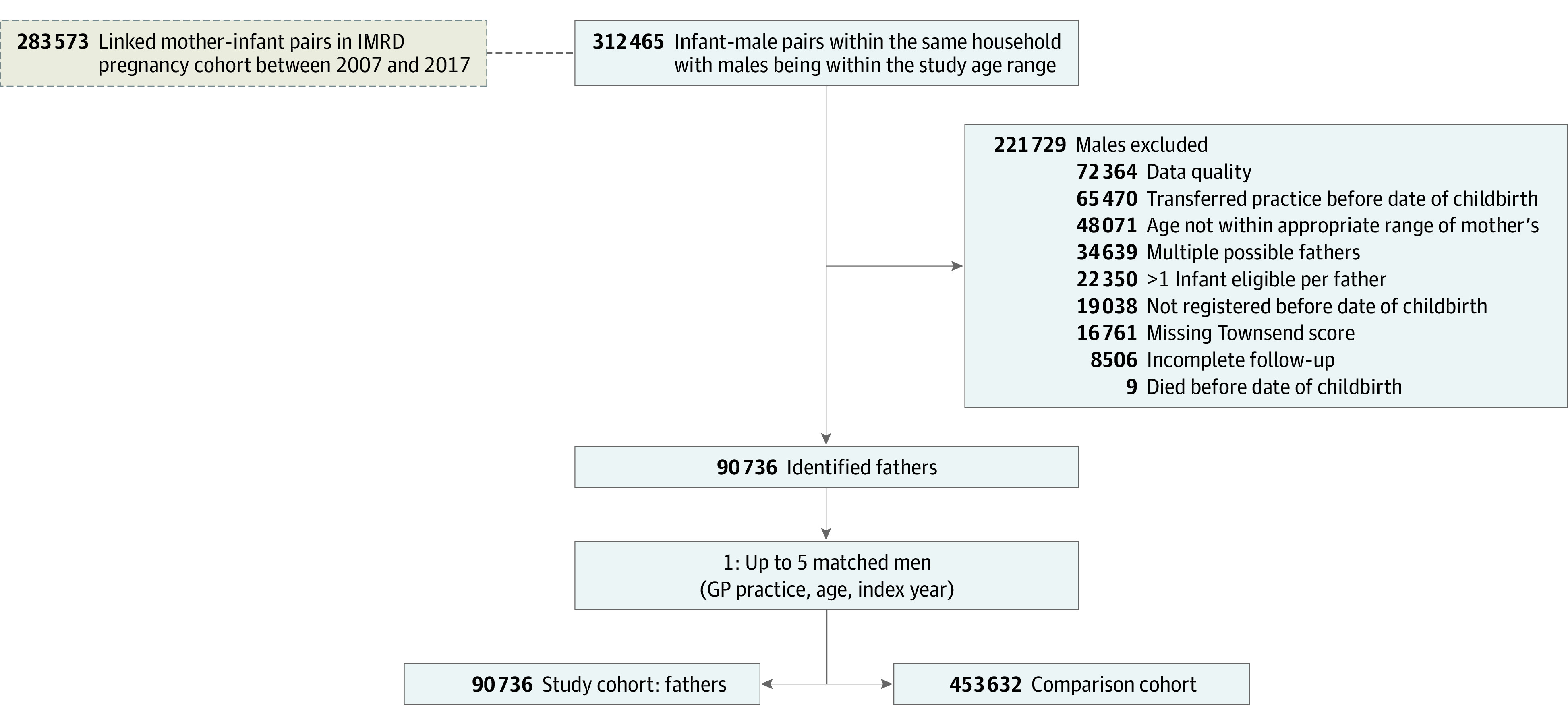
Flowchart of Study Recruitment With Inclusion and Exclusion Criteria GP indicates general practitioner.

### Paternal Characteristics

Most men (463 879 men [85.2%]) were aged between 25 and 44 years, and there were more men living in the least deprived areas (130 277 men [23.9%]) than most deprived areas (72 268 men [13.3%]) (Table 1), which represents the overall distribution of this database.^13^ Fathers were less likely to have a recent history of antidepressant treatment than men in the comparison cohort (3840 men [4.2%] vs 26 109 men [5.8%]) and likewise were less likely to have previous treatment (1206 men [1.3%] vs 7516 men [1.7%]). Overall, 5.5% of fathers had some history of antidepressant treatment, compared with 7.5% of men in the comparison cohort. The database included more individuals in 2007 to 2008 than in later years; hence, a larger proportion of the men in this cohort were from these years (Table 1).

**Table 1.** Characteristics of Men at Index Date, Comparing Fathers and Men in the Comparison Cohort

Characteristic	Participants, No. (%)	
	Fathers (n = 90 736)	Comparison cohort (n = 453 632)
Age, y		
15-19	2984 (3.3)	14 920 (3.3)
20-24	6111 (6.7)	30 550 (6.7)
25-29	13 447 (14.8)	67 231 (14.8)
30-34	25 951 (28.6)	129 731 (28.6)
35-39	25 525 (28.1)	127 619 (28.1)
40-44	12 397 (13.7)	61 978 (13.7)
45-49	3606 (4.0)	18 028 (4.0)
50-54	715 (0.8)	3575 (0.8)
Townsend Score quintile		
1 (least deprived)	22 803 (25.1)	107 474 (23.7)
2	19 216 (21.2)	92 096 (20.3)
3	20 195 (22.3)	102 043 (22.5)
4	17 312 (19.1)	90 961 (20.1)
5 (most deprived)	11 210 (12.4)	61 058 (13.5)
History of antidepressant treatment		
Recent	3840 (4.2)	26 109 (5.8)
Previous	1206 (1.3)	7516 (1.7)
None	85 690 (94.4)	420 007 (92.6)
Year group		
2007-2008	23 233 (25.6)	116 150 (25.6)
2009-2010	20 968 (23.1)	104 825 (23.1)
2011-2012	19 112 (21.1)	95 551 (21.1)
2013-2014	16 606 (18.3)	83 021 (18.3)
2015-2016	10 736 (11.9)	54 085 (11.9)

### Factors Associated With Antidepressant Treatment in Fathers

Overall, 4439 fathers (4.9%) had an antidepressant prescription in the year after childbirth, compared with 26 646 men (5.9%) who did not have a child in the same year (Table 2). Likelihood of postnatal antidepressant treatment in fathers increased with deprivation (eg, most deprived: PPR vs least deprived, 1.79; 95% CI, 1.62-1.99), and this difference remained significant for the fathers living with the most deprivation after considering age, history of antidepressant treatment and calendar year in a fully adjusted model (adjusted PRR, 1.18; 95% CI, 1.07-1.30) (Table 3). Compared with no history of antidepressant treatment, history of antidepressant treatment was strongly associated with a postnatal antidepressant prescription, with those with a recent prescription being more than 30 times as likely to have a postnatal prescription (adjusted PRR, 32.31; 95% CI, 30.37-34.38) history and those with a previous prescription, but no recent prescription being 7 times as likely to have a postnatal prescription (adjusted PRR, 7.04; 95% CI, 6.03-8.23). In the fully adjusted model, likelihood of a postnatal antidepressant prescription was similar across age categories and year group (Table 3).

**Table 2.** Proportion of Men With an Antidepressant Prescription in the Year After Having a Child or Equivalent Index Date Among Fathers and the Comparison Cohort, by Characteristic

Characteristic	Fathers		Comparison cohort	
	No.	Antidepressant prescription, No. (%)	No.	Antidepressant prescription, No. (%)
Overall	90 736	4439 (4.9)	453 632	26 646 (5.9)
Age, y				
15-19	2984	76 (2.6)	14 920	315 (2.1)
20-24	6111	387 (6.3)	30 550	1503 (4.9)
25-29	13 447	785 (5.8)	67 231	3611 (5.4)
30-34	25 951	1118 (4.3)	129 731	7409 (5.7)
35-39	25 525	1208 (4.7)	127 619	7986 (6.3)
40-44	12 397	624 (5.0)	61 978	4309 (7.0)
45-49	3606	200 (5.6)	18 028	1241 (6.9)
50-54	715	41 (5.7)	3575	272 (7.6)
Townsend Score quintile				
1 (least deprived)	22 803	906 (4.0)	107 474	5081 (4.7)
2	19 216	765 (4.0)	92 096	4607 (5.0)
3	20 195	982 (4.9)	102 043	5740 (5.6)
4	17 312	988 (5.7)	90 961	5957 (6.6)
5 (most deprived)	11 210	798 (7.1)	61 058	5261 (8.6)
History of antidepressant treatment				
Recent	3840	2552 (66.5)	26 109	17 289 (66.2)
Previous	1206	175 (14.5)	7516	1042 (13.9)
None	85 690	1712 (2.0)	420 007	8315 (2.0)
Year group				
2007-2008	23 233	947 (4.1)	116 150	5805 (5.0)
2009-2010	20 968	986 (4.7)	104 825	5646 (5.4)
2011-2012	19 112	969 (5.1)	95 551	5910 (6.2)
2013-2014	16 606	933 (5.6)	83 021	5523 (6.7)
2015-2016	10 736	604 (5.6)	54 085	3762 (7.0)

**Table 3.** Mixed-Effects Poisson Estimates of the Likelihood of Having a Postnatal Antidepressant Prescription in Fathers Only, by Characteristic

Characteristic	Model, PRR (95% CI)^a^		
	Unadjusted	Age, deprivation, and year group	Fully adjusted^b^
Age, y			
15-19	0.54 (0.42-0.68)	0.49 (0.39-0.62)	0.83 (0.66-1.05)
20-24	1.34 (1.19-1.51)	1.25 (1.11-1.40)	1.12 (1.00-1.26)
25-29	1.28 (1.17-1.41)	1.22 (1.11-1.34)	1.14 (1.04-1.25)
30-34	1 [Reference]	1 [Reference]	1 [Reference]
35-39	1.13 (1.04-1.22)	1.16 (1.07-1.25)	1.07 (0.99-1.16)
40-44	1.20 (1.09-1.33)	1.23 (1.11-1.36)	1.05 (0.95-1.16)
45-49	1.32 (1.14-1.54)	1.34 (1.15-1.56)	1.07 (0.92-1.24)
50-54	1.36 (0.99-1.86)	1.36 (1.00-1.86)	1.11 (0.81-1.51)
Townsend Score quintile			
1 (least deprived)	1 [Reference]	1 [Reference]	1 [Reference]
2	1.01 (0.92-1.11)	1.01 (0.92-1.12)	0.96 (0.87-1.06)
3	1.25 (1.14-1.37)	1.26 (1.14-1.38)	1.04 (0.95-1.14)
4	1.46 (1.33-1.61)	1.48 (1.35-1.63)	1.08 (0.99-1.18)
5 (most deprived)	1.79 (1.62-1.99)	1.82 (1.64-2.02)	1.18 (1.07-1.30)
History of antidepressant treatment			
Recent	33.26 (31.29-35.36)	NA	32.31 (30.37-34.38)
Previous	7.26 (6.22-8.49)	NA	7.04 (6.03-8.23)
None	1 [Reference]	NA	1 [Reference]
Year group			
2007-2008	1 [Reference]	1 [Reference]	1 [Reference]
2009-2010	1.15 (1.05-1.26)	1.14 (1.04-1.25)	1.08 (0.99-1.19)
2011-2012	1.24 (1.13-1.36)	1.23 (1.12-1.34)	1.10 (1.01-1.21)
2013-2014	1.38 (1.26-1.51)	1.36 (1.24-1.49)	1.12 (1.02-1.23)
2015-2016	1.36 (1.22-1.51)	1.32 (1.19-1.47)	1.04 (0.94-1.15)

Abbreviations: NA, not applicable; PRR, prevalence rate ratio.

^a^ Practice is included as random effects terms.

^b^ Adjusted for age, social deprivation, history of antidepressant treatment, and year group.

### Fatherhood and Antidepressant Treatment

Of the men in our comparison cohort, 26 646 (5.9%) had an antidepressant prescription in the follow-up period compared with 4.9% of fathers (Table 2). In unadjusted analysis, fathers were 17% less likely to have an antidepressant prescription compared with men in the comparison cohort in the year after childbirth or equivalent follow-up period (PRR, 0.83, 95% CI, 0.81-0.86); after adjusting for age, deprivation, and year group, fathers were 15% less likely to have an antidepressant prescription (adjusted PRR: 0.85; 95% CI, 0.82-0.87). However, once history of antidepressant treatment was included in the fully adjusted model, there was no difference in antidepressant prescriptions between groups (adjusted PRR, 1.01; 95% CI, 0.98-1.04) (eAppendix and eTable 2 in Supplement 1). Men in the comparison cohort were initiated on antidepressant treatment earlier: the median (IQR) time to first antidepressant prescription from date of childbirth or index date was 59 (20-185) days for fathers and 40 (16-142) days for men in the comparison cohort.

## Discussion

This comparative cohort study found that recent fathers were 17% less likely to be prescribed antidepressants in the year after having a child compared with men who did not have a child in the same year, which was contrary to our hypothesis. However, once men’s prior history of antidepressant treatment was accounted for, we found no difference between these cohorts. In fathers, the likelihood of having an antidepressant prescription increased with deprivation, and having a history of antidepressant treatment was strongly associated with having a prescription after having a child; these associations persisted after adjusting for all other factors. Our cohorts were similar on matched variables, such as age and calendar year; however, there was an underrepresentation of the most deprived groups in our father cohort, but this is consistent with the overall distribution of this database. Our findings suggest that it is essential to understand previous and recent antidepressant treatment and deprivation when identifying men in need of assessment and potentially further treatment after having a child.

To our knowledge, this is the first study to investigate antidepressant treatment in recent fathers and a comparative cohort of men using the same data source and methods. Our sample of fathers was large, at more than 90 000 men, and we were able to draw on a large population database for our comparison cohort of more than 450 000 men, which adds robustness to our findings.

We identified no previous studies investigating antidepressant treatment in recent fathers; however, a 2019 meta-analysis conducted by Rao et al^9^ estimated, based on 47 studies, that 8.8% of men experienced depression in the year after having a child. Rao et al^9^ included studies in which depression was identified using a validated tool, the Edinburgh Postnatal Depression Scale (EPDS), using a survey design. In our study, we found that 4.9% of fathers had an antidepressant prescription in the year after having a child. We would expect the use of antidepressants to be lower than the estimate of depression, as our study included men who engaged with primary care services and sought and received pharmacological treatment for their depression, which is likely to be a subset of all men experiencing depression who would be identified in survey studies.

### Implications of Findings and Future Research Recommendations

While we identified a lower proportion of fathers with an antidepressant prescription compared with men in the comparison cohort, this could be attributed to a difference in history of antidepressant treatment between groups. Our father cohort had a lower proportion of men with any past treatment compared with men in the comparison cohort (5.5% vs 7.5%), and once we fully accounted for this difference in our analysis, we saw no difference in antidepressant prescriptions in the follow-up period between our cohorts.

There remains much stigma for men to receive mental health treatments, particularly at a time when there is a lot of focus on the health of the baby and mother. As such, fathers may not be identified as a priority after the birth of a child. This may be due to their own choices, priorities in the family unit, or limitations in the broader health care system. While past emphasis has been on the postnatal check for mothers,^17^ fathers potentially at higher risk of needing antidepressant treatment identified in this study (ie, those living in more deprived areas and those with a history of antidepressant treatment) could be invited for their own mental health check-up after having a child. The benefits of such an intervention could be explored in future studies. As we found a higher proportion of men treated with antidepressant in those who did not have a newborn child in the previous year than in those who did, it is possible that men treated with an antidepressant were less likely to become fathers. Further research is needed to investigate whether using antidepressants or experiencing depression can be a barrier to fatherhood.

### Limitations

This study has some limitations. The main limitation of our study is being able to correctly identify fathers within our data set, as the method of identification relies on women of a comparable age within the same household and having a record of a baby within the study window. We have no definitive way of confirming that those identified as fathers in our study were fathers. Furthermore, there are some fathers who will be missed, as some partners may not live together, may not be registered with the same GP, or may live in larger households with multiple eligible men, making it challenging to identify the father. As such, we will likely underestimate the number of fathers within the data set, but this is unlikely to affect the direction of our findings.

In addition, in this study, we focused on the men who were prescribed antidepressants but did not examine whether they had symptoms or diagnoses of depression. However, it is possible that some men might not meet the threshold for pharmacological treatment or not want antidepressants. Thus, our findings may underestimate the prevalence of depression among men who have recently had children.

## Conclusion

This cohort study found that recent fatherhood was not associated with an increase in antidepressant treatment. However, previous antidepressant treatment and social deprivation were key determinants associated with antidepressant treatment in the year after having a child. Further research is needed to determine whether antidepressant treatment or experiencing depression can be a barrier to fatherhood and whether recent fathers experience barriers to antidepressant treatment in turn.

## Notes

Supplement 1. **eAppendix.** Inclusion and Exclusion Criteria Used to Identify Fathers **eTable 1.** Exploration of Different Time Periods Relating to History of Antidepressant Treatment for Fathers and Men in the Comparison Cohort **eTable 2.** Mixed-Effects Poisson Estimates of the Likelihood of Having a Postnatal Antidepressant Prescription Comparing Fathers and Men in the Comparison Cohort

Supplement 2.
Data Sharing Statement
