# Supplementary material for: Association of Recent Fatherhood With Antidepressant Treatment Initiation Among Men in the United Kingdom
Source: JAMA Netw Open. 2023 May 31;6(5):e2316105. doi: 10.1001/jamanetworkopen.2023.16105 (PMC10233418; doi:10.1001/jamanetworkopen.2023.16105)
Supplement: Supplement 1. — eAppendix. Inclusion and Exclusion Criteria Used to Identify Fathers eTable 1. Exploration of Different Time Periods Relating to History of Antidepressant Treatment for Fathers and Men in the Comparison Cohort eTable 2. Mixed-Effects Poisson Estimates of the Likelihood of Having a Postnatal Antidepressant Prescription Comparing Fathers and Men in the Comparison Cohort [file jamanetwopen-e2316105-s001.pdf]

Supplemental Online Content

Smith HC, Petersen I, Schartau P. Association of recent fatherhood with antidepressant treatment initiation among men in the United Kingdom. *JAMA Netw Open*. 2023;6(5):e2316105. doi:10.1001/jamanetworkopen.2023.16105

eAppendix. Inclusion and Exclusion Criteria Used to Identify Fathers
eTable 1. Exploration of Different Time Periods Relating to History of Antidepressant Treatment for Fathers and Men in the Comparison Cohort
eTable 2. Mixed-Effects Poisson Estimates of the Likelihood of Having a Postnatal Antidepressant Prescription Comparing Fathers and Men in the Comparison Cohort

This supplemental material has been provided by the authors to give readers additional information about their work.

© 2023 Smith HC et al. *JAMA Network Open.*

eAppendix. Inclusion and Exclusion Criteria Used to Identify Fathers

Men who were not registered at a practice before their index date – infant's date of birth – were excluded. We excluded potential fathers where the age gap between cohabitant men and women was more than 15 years, as this could indicate a different male living at the same address (such as a grandparent or other child) rather than a potential father. In addition, for men aged 18 years or younger at date of childbirth we excluded those where the age gap with cohabiting women was more than 10 years as this could indicate a mother (aged 30 years) and son (aged 15 years). Men who didn't have complete follow- up information up to their child turning one year of age (i.e. they died or transferred practice) and/or were missing social deprivation information were excluded. Where multiple potential fathers met the above criteria for the same infant, all were excluded from the study as it was not possible to determine who the likely father was. These cases could indicate a household covering multiple families at one address, such as a block of flats. Where men had multiple eligible children within the study period, one was selected at random for inclusion.

© 2023 Smith HC et al. *JAMA Network Open.*

eTable 1. Exploration of Different Time Periods Relating to History of Antidepressant Treatment for Fathers and Men in the Comparison Cohort

These tables present conditional frequency of records: given that one meets the criteria on a given row on the left-hand side, what is the frequency of meeting the criteria across the columns along the top. For example, the table illustrates, in fathers, that 40% of those in E, also met the criteria for A. On the other hand, 34% of those in A, also met the criteria for E. These correspond to: A – Individuals who were prescribed at least 1 antidepressant more than 2 years before delivery. B – Individuals who were prescribed at least 1 antidepressant 2 years before delivery up to 1 year before delivery. C – Individuals who were prescribed at least 1 antidepressant in year prior to delivery. D – Individuals who were prescribed at least 1 antidepressant in the 18 to 6 months before start of pregnancy (27months-15 months before delivery). E – Individuals who were prescribed at least 1 antidepressant in the 1 year after delivery. Here, delivery refers to the father's child's date of birth or equivalent index date for those in the comparison cohort

Fathers, N=90,736	A	B	C	D	E
A	A	59%	47%	69%	40%
B	31%	B	57%	91%	41%
C	28%	64%	C	59%	57%
D	27%	54%	66%	D	38%
E	34%	85%	49%	53%	E

Comparison cohort, N=453,632	A	B	C	D	E
A	A	69%	55%	75%	51%
B	31%	B	57%	91%	46%
C	29%	67%	C	62%	65%
D	28%	55%	66%	D	43%
E	33%	88%	51%	53%	E

© 2023 Smith HC et al. *JAMA Network Open.*

eTable 2. Mixed-Effects Poisson Estimates of the Likelihood of Having a Postnatal Antidepressant Prescription Comparing Fathers and Men in the Comparison Cohort

	Models, PRR (95% CI)		
Characteristic	Unadjusted	Age, deprivation & year group	Fully adjusted
Cohort			
Fathers	0.83 (0.81-0.86)	0.85 (0.82-0.87)	1.01 (0.98-1.04)
Comparison cohort	1	1	1
Paternal age (years)			
15-19	0.34 (0.31-0.38)	0.34 (0.31-0.38)	0.76 (0.69-0.84)
20-24	0.82 (0.78-0.87)	0.81 (0.77-0.85)	0.96 (0.91-1.01)
25-29	0.92 (0.88-0.95)	0.91 (0.87-0.94)	0.97 (0.94-1.01)
30-34	1	1	1
35-39	1.14 (1.11-1.18)	1.17 (1.13-1.20)	1.06 (1.03-1.09)
40-44	1.27 (1.22-1.31)	1.30 (1.26-1.35)	1.09 (1.05-1.12)
45-49	1.28 (1.21-1.36)	1.31 (1.24-1.57)	1.10 (1.04-1.16)
50-54	1.38 (1.23-1.55)	1.40 (1.25-1.57)	1.15 (1.02-1.28)
Townsend Score quintile			
1-least deprived	1	1	1
2	1.06 (1.02-1.10)	1.06 (1.02-1.10)	0.99 (0.96-1.03)
3	1.25 (1.20-1.29)	1.25 (1.21-1.30)	1.05 (1.01-1.08)
4	1.46 (1.41-1.52)	1.48 (1.43-1.54)	1.11 (1.07-1.15)
5-most deprived	1.89 (1.82-1.97)	1.93 (1.85-2.01)	1.21 (1.16-1.26)
History of antidepressant treatment			
Recent	33.12 (32.32-33.93)		32.16 (31.37-32.96)
Previous	6.98 (5.58-7.41)		6.83 (6.43-7.25)
None	1		1
Year group			
2007-2008	1	1	1
2009-2010	1.09 (1.05-1.13)	1.09 (1.06-1.13)	1.04 (1.01-1.08)
2011-2012	1.24 (1.20-1.28)	1.25 (1.20-1.29)	1.09 (1.06-1.13)
2013-2014	1.33 (1.28-1.38)	1.34 (1.29-1.38)	1.10 (1.06-1.14)
2015-2016	1.34 (1.28-1.39)	1.34 (1.29-1.39)	1.02 (0.99-1.07)

Abbreviations: PRR – prevalence rate ratio, CI – confidence interval.
Practice is included as random effects terms
