# Supplementary material for: Association of Recent Fatherhood With Antidepressant Treatment Initiation Among Men in the United Kingdom
Source: JAMA Netw Open. 2023 May 31;6(5):e2316105. doi: 10.1001/jamanetworkopen.2023.16105 (PMC10233418; doi:10.1001/jamanetworkopen.2023.16105)
Supplement: Supplement 2. — Data Sharing Statement [file jamanetwopen-e2316105-s002.pdf]

Data Sharing Statement

Smith. Association of Recent Fatherhood With Antidepressant Treatment Initiation Among Men in the United Kingdom. *JAMA Netw Open*. Published May 31, 2023. doi:10.1001/jamanetworkopen.2023.16105

Data
Data available: No

Additional Information
Explanation for why data not available: No additional data are available as this work draws on de-identified data provided by patients as a part of their routine primary care.
